# Significant up-regulation of lncRNAs in neuromyelitis optica spectrum disorder

**DOI:** 10.1038/s41598-023-45457-w

**Published:** 2023-10-31

**Authors:** Mohammad Taheri, Ahmad Sadeghi, Alireza Gharebaghi, Masoud Ghiasian, Solat Eslami, Sheyda Khalilian, Arezou Sayad, Soudeh Ghafouri-Fard

**Affiliations:** 1 Institute of Human Genetics, Jena University Hospital, Jena, Germany; 2 Urology and Nephrology Research Center, Shahid Beheshti University of Medical Sciences, Tehran, Iran; 3 Department of Medical Genetics, Shahid Beheshti University of Medical Sciences, Tehran, Iran; 4 Neurophysiology Research Center, Hamadan University of Medical Sciences, Hamadan, Iran; 5 Department of Neurology, Hamadan University of Medical Sciences, Hamadan, Iran; 6 Dietary Supplements and Probiotic Research Center, Alborz University of Medical Sciences, Karaj, Iran; 7 Department of Medical Biotechnology, School of Medicine, Alborz University of Medical Sciences, Karaj, Iran

**Keywords:** Genetics, Neurology

## Abstract

Neuromyelitis optica spectrum disorder (NMOSD) is an immune-related demyelinating defect. Long non-coding RNAs (lncRNAs) might influence the pathobiology and progression of NMOSD. The current study assessed expression level of NEAT1, PANDAR, MEG3 and TUG1 lncRNAs in the peripheral blood of NMOSD patients compared with healthy individuals. All mentioned lncRNAs were shown to be over-expressed in total NMOSD cases, male NMOSD cases and female NMOSD cases compared with the matching control subgroups. MEG3 had the most robust over-expression in patients subgroups compared with normal subjects. There was no noteworthy difference in the expression of any of lncRNAs between female and male patients. MEG3 had an ideal performance in the differentiation of NMOSD cases from healthy persons (Sensitivity and specificity values = 100%). Other lncRNAs could also efficiently separate NMOSD cases from control subjects (AUC values = 0.97, 0.89 and 0.88 for PANDAR, NEAT1 and TUG1, respectively). Cumulatively, NEAT1, PANDAR, MEG3 and TUG1 lncRNAs can be considered as appropriate disease markers for NMOSD.

## Introduction

Neuromyelitis optica spectrum disorder (NMOSD) is an immune-related demyelinating defect of the central nervous system (CNS) characterized by the optic nerve and spinal cord disorders^1–3^. NMOSD lesions also cause frequent brain attacks by involving the brain stem. These attacks appear as optic neuritis, myelitis and some brainstem disorders^4^. NMOSD is a severe debilitating disorder that can lead to irreversible consequences, including everlasting blindness, paralysis or even demise^5^. NMOSD patients usually have severe continuous neuropathic pain. In some patients, severe pain affects the quality of life^6^. According to epidemiological studies, NMOSD is a rare disorder with a worldwide prevalence of 0.3–0.5–4.4 in 100,000 population^7^. NMOSD is often prompted by IgG autoantibodies against aquaporin 4 (AQP4)^8^. Nowadays, treatment with steroids and plasma exchange along with immunosuppressants are applied to improve the long-term quality of life. However, many NMOSD patients still have a poor prognosis^9^.

Long non-coding RNAs (lncRNAs) are considered as the chief regulators of gene expression^10^. These transcripts act by binding to histone modifying complexes, transcription factors and RNA polymerase II^11^. Dysregulated expression of lncRNAs has been associated with the immunological imbalance^12^. According to the previous studies, lncRNAs play an imperative role in autoimmune diseases since they are involved in the differentiation and activity of T and B cells, macrophages and natural killer cells^13^. Numerous researches have shown aberrant expression of lncRNAs in the peripheral blood of patients with NMOSD^14–16^. However, the function of some other lncRNAs in the pathobiology of this demyelinating inflammatory disease has not been investigated.

Generally, NMOSD has been suggested to be an Asian optic-spinal kind of multiple sclerosis (MS), and has some pathologic similarities with MS. In addition, the cytokine profile is similar between MS and NMOSD^17^. MS, as an immunological defect of the CNS, has motivated researchers to investigate the lncRNAs expression profile in relation to the disease phenotype^18^. Recently, we evaluated the expression level of NEAT1, PANDA and TUG1 in the blood of MS patients compared with healthy individuals. Our results demonstrated that the expression of all three lncRNAs have been significantly increased in MS patients^19^.

NEAT1 regulates the innate immune responses via stimulation of the expression of IL-8^20^. PANDAR and TUG1 are lncRNAs that are functionally associated with P53^21,22^. Evidence has also demonstrated an increase in the expression of NEAT1, TUG1, and PANDAR in interferon-β-responsive MS population compared with the healthy individuals. In addition, TUG1 and NEAT1 expressions were inversely correlated with onset age and disease duration in female MS patients^23^. NEAT1 and TUG1 over-expressions were also reported in Italian MS patients in comparison with healthy controls^18^.

In relation to the shared features between MS and NMOSD, in the current study, we have focused on the above lncRNAs to measure their expression in the blood of NMOSD patients and controls. Besides, the expression level of an imprinted gene, namely maternally expressed gene 3 (MEG3) that is involved in the immune system and the MS development^24^ is assessed in the present study. MEG3 plays important roles in various cancers as a tumor suppressor^25^. Additionally, the roles of MEG3 in the modulation of immune response are also suggested^23^. For example, during bacterial infection, MEG3 has been reported to exacerbate inflammatory responses and subsequently increase IL-1β^26^.

Accordingly, deregulation of the chosen lncRNAs might be involved in the NMOSD pathobiology or used as disease markers.

## Material and methods

### Patients and controls

Expression of NEAT1, TUG1, MEG3 and PANDAR lncRNAs was assessed in blood samples of patients with NMOSD and matched controls. Patients were admitted to hospitals affiliated to Shahid Beheshti University of Medical Sciences. All of patients were AQP4 antibody positive. None of them received immunosuppressive treatment during a period of one month before sampling. The study protocol was approved by the ethical committee of Shahid Beheshti University of Medical Sciences. Informed consent forms were obtained from all NMOSD cases and controls.

### Expression assay

Total RNA was extracted from blood samples using RNJia extraction kit (Roje Technologies Company, Iran). Afterwards, cDNA was made using AddScript cDNA Synthesis Kit (ADDBIO, South Korea). Expression of lncRNAs was determined using RealQ Plus 2× Master Mix (AMPLIQON, Denmark). Primers were designed by authors and purchased from the METABION Company (Germany). PCR conditions and primer sequences were the same as our previous papers^19,27^. Table S1 shows the primers sequences.

### Statistical analysis

Data was analyzed using GraphPad Prism version 9.0 (La Jolla, CA, USA). We compared the expression levels of four genes, namely MEG3, TUG1, NEAT1 and PANDAR in NMOSD patients and healthy controls. Expression amounts were calculated using the comparative –delta Ct method. Shapiro–wilk test was used for assessment of distribution of data values. Mann–Whitney U test was used to report differential expression of genes between NMOSD patients and healthy controls. Two-way ANOVA and Tukey post hoc tests were used to evaluate the effects of disease and gender on gene expression levels. Correlations between lncRNAs expression levels were calculated using Spearman’s rank correlation coefficient since expression data was not normally distributed. Also, correlations between gene expression levels and clinical parameters such as age at onset and EDSS scores were measured with Spearman’s rank correlation coefficient.

The receiver operating characteristic (ROC) curves were depicted to appraise the diagnostic ability of differentially expressed genes.

### Ethics approval and consent to participate

All procedures performed were in accordance with the ethical standards of the national research committee and with the 1964 Helsinki declaration and its later amendments. Informed consent forms were obtained from all study participants. The study protocol was approved by the ethical committee of Shahid Beheshti University of Medical Sciences.

## Results

### General demographic data

General information about NMOSD cases is shown in Table 1. Sex ratios (male/female) in cases and controls were 10/32 and 9/39 (P value = 0.55).

**Table 1:** General data of NMOSD cases.

Data	Groups	
Sex (number)	Male	10
	Female	32
Age (mean ± SD)	Male	39.9 ± 16.52
	Female	37.62 ± 10.12
Disease duration (mean ± SD)	Male	2.6 ± 1.34
	Female	2.96 ± 1.37
Age of onset (mean ± SD)	Male	37.3 ± 15.76
	Female	34.65 ± 9.7
EDSS Start Score	Male	2 ± 2.46
	Female	2.37 ± 1.43
EDSS Current Score	Male	2 ± 2.21
	Female	2.4 ± 1.58

In addition, control group included 9 male (mean age ± SD: 36.11 ± 13.89) and 39 female healthy subjects (mean age ± SD: 40.69 ± 10.91). There was no significant difference in age between cases and controls (P values = 0.59 and 0.22 for males and females, respectively).

### Expression assays

**Figure 1:**
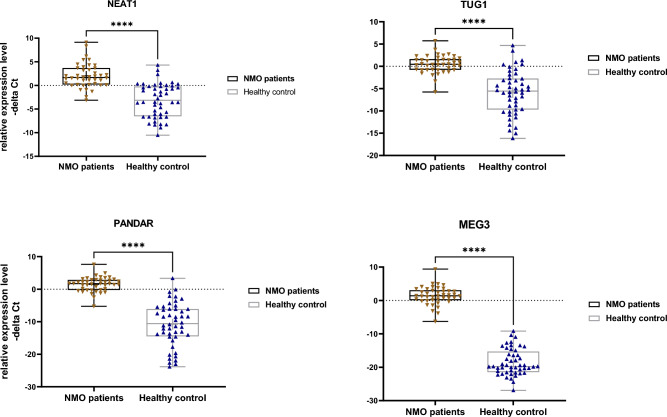
Expression level of four genes in neuromyelitis optica (NMO) patients (total) and controls (total) as described by –delta Ct values (**A–E**). Mann–Whitney U test was used (****P value < 0.0001).

Experiments in the peripheral blood samples revealed significant up-regulation of PANDAR, NEAT1, TUG1 and MEG3 lncRNAs in NMOSD cases compared with controls (Fig. 1).

**Figure 2:**
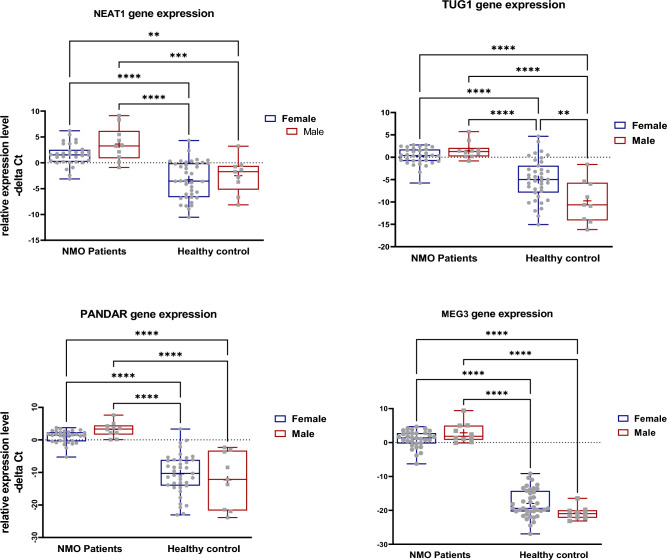
Expression levels of four studied genes in neuromyelitis optica (NMO) patients’ subgroups (males and females) versus control subgroups (males and females) as described by –delta Ct values. Two-way ANOVA and Tukey post hoc tests were used to analyze data (*P value < 0.05, **P value < 0.001 and ****P value < 0.0001).

Afterwards, we analyzed expression data based on the gender of study participants (Fig. 2). Expressions of mentioned lncRNAs were also higher in both male and female NMOSD patients compared with the matched controls.

Group (disease) factor had a significant effect of on the expression level of all four lncRNAs. However, gender had no important effect on expression levels of any of studied lncRNAs. The interaction of gender and group showed a significant effect on expression of TUG1 and MEG3 lncRNA genes (Table 2).

**Table 2:** Analysis of effect of group and gender (tests of between-subjects effects) on expression of four studied genes in NMOSD cases compared with healthy controls.

Source of Variation	Group effect (disease)			Gender effect			Interaction		
	SS	F	P value	SS	F	P value	SS	F	P value
NEAT1	445	46.72	** < 0.0001**	30.85	3.23	0.07	6.26	0.65	0.42
TUG1	1011	78.13	** < 0.0001**	43.13	3.13	0.07	140.1	10.82	**0.0015**
PANDAR	2687	105	** < 0.0001**	0.56	0.02	0.88	62.2	2.5	0.12
MEG3	6789	616	** < 0.0001**	3.1	0.28	0.59	81.72	7.42	**0.0078**

*SS* sum of squares, *F *F of variance.

Significant values are in bold.

All mentioned lncRNAs were shown to be over-expressed in total, male as well as female NMOSD cases compared with the consistent control subgroups. MEG3 had the most robust over-expression in patients subgroups compared with controls (Table 3 There was no significant difference in the expression of any of lncRNAs between female and male patients (Table 3).

**Table 3:** The results of expression ratio (fold change) of four studied genes in patients with NMOSD cases compared to healthy controls.

		Total cases vs. controls (42 vs. 48)	Male cases vs. male controls (10 vs. 9)	Female cases vs. female controls (32 vs. 9)	Female cases vs. male patients (32 vs. 10)
NEAT1	ER (95% CI)	44 (14.6–128)	69 (5.24–910)	28 (7.36–910)	0.23 (0.02–1.8)
	APV	< 0.0001	< 0.0001	< 0.0001	0.25
TUG1	ER (95% CI)	300 (83.2–1024)	2521 (124–49,667)	35.7 (7.5–170)	0.36 (0.03–4.14)
	APV	< 0.0001	< 0.0001	< 0.0001	0.72
PANDAR	ER (95% CI)	10,809 (1782–65,536)	45,053 (657–3,070,410)	2665 (296–23,821)	0.21 (0.007–5.9)
	APV	< 0.0001	< 0.0001	< 0.0001	0.61
MEG3	ER (95% CI)	2,097,152 (794,672–8,388,608)	13,346,887 (834,180–203,436,033)	489,178 (122,294–2,186,209)	0.27 (0.03–2.39)
	APV	< 0.0001	< 0.0001	< 0.0001	0.4

Two-way ANOVA and Tukey post hoc tests were used to analyze the data (*ER* expression ratio, *APV* adjusted P value).

The strongest correlations were detected between TUG1 and PANDAR (correlation coefficient = 0.9) and between TUG1 and MEG3 (Correlation coefficient = 0.8) in NMOSD cases (Table 4).

**Table 4:** Spearman’s correlations between expression levels of lncRNAs among the NMOSD patients and controls.

	TUG1		PANDAR		MEG3	
	Patients	Controls	Patients	Controls	Patients	Controls
NEAT1	0.63**	0.33*	0.69**	0.3*	0.35*	0.25*
TUG1			0.9**	−0.02	0.8**	0.37*
PANDAR					0.74**	0.28*

*p < 0.05.

**p < 0.001.

**Figure 3:**
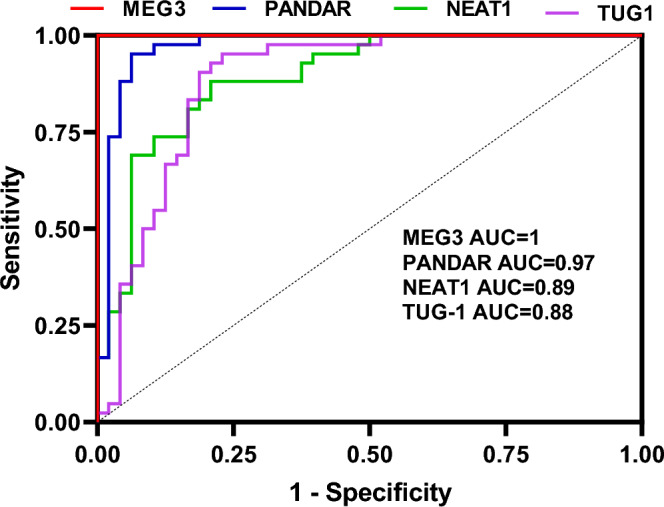
ROC curves of transcript levels of lncRNAs in patients with NMOSD.

MEG3 had an ideal performance in the separation of NMOSD cases from healthy controls (Sensitivity and specificity values = 100%). Other lncRNAs could also efficiently separate NMOSD cases from controls (AUC values = 0.97, 0.89 and 0.88 for PANDAR, NEAT1 and TUG1, respectively) (Fig. 3 and Table 5).

**Table 5:** The results of ROC curve analysis for four differentially expressed genes in patients with NMOSD.

MEG3				PANDAR				NEAT1				TUG1			
AUC ± SD	Sensitivity	Specificity	P Value	AUC ± SD	Sensitivity	Specificity	P Value	AUC ± SD	Sensitivity	Specificity	P Value	AUC ± SD	Sensitivity	Specificity	P Value
1 ± 0.000	1	1	< 0.0001	0.97 ± 0.01	0.95	0.93	< 0.0001	0.89 ± 0.03	0.88	0.79	< 0.0001	0.88 ± 0.03	0.95	0.77	< 0.0001

TUG1 and PANDAR expression levels were positively correlated with age and age at onset. Moreover, NEAT1 expression level was negatively correlated with EDSS at the start point (Table 6).

**Table 6:** Spearman’s rank correlation between expressions of four lncRNAs, and demographic/clinical parameters, including age, sex, disease duration, age at onset and EDSS scores.

	Age	Sex	Disease duration	Age at onset	EDSS start	EDSS current
NEAT	0.042	0.277	0.062	0.032	−0.322*	−0.167
TUG1	0.368*	0.226	0.109	0.355*	−0.099	−0.058
PANDAR	0.351*	0.434**	0.017	0.351*	−0.194	−0.096
MEG3	0.232	0.212	−0.134	0.246	0.103	0.071
Age		0.039	0.415**	0.995**	0.058	0.038
Sex			−0.141	0.046	−0.258	−0.158
Disease duration				0.353*	0.115	0.187
Age onset					0.050	0.027
EDSS start						0.408**

EDSS Scores were classified into 2 ranges (1–2 and greater than 2).

*p < 0.05.

**p < 0.001.

## Discussion

NMOSD is an immune-related demyelinating defect of the CNS with several shared features with MS. Dysregulation of several types of RNAs, including non-coding RNAs has been reported in the peripheral blood mononuclear cells of these patients^28^. The results of the current study revealed substantial up-regulation of NEAT1, PANDAR, MEG3 and TUG1 in NMOSD patients compared with healthy individuals. Notably, MEG3 had the most robust over-expression in patients subgroups compared with controls. Not surprisingly, NEAT1, PANDAR and TUG1 have also been reported to be over-expressed in MS patients compared with healthy subjects^19^. However, MEG3 has been reported to be reduced in the relapse phase of MS compared to remission phase and healthy groups^24^. The results of the latter study has also been confirmed in another study showing down-regulation of MEG3 in both female and male MS patients compared with matched control subjects^29^. Thus, MEG3 can be considered as a differentiating lncRNA between MS and NMOSD. Other lncRNAs may just indicate the presence of an autoimmune condition, not differentiating between MS and NMOSD. The relation between expression levels of MEG3 and immune responses has been confirmed in a study in brain tumors. Notably, in glioblastoma multiforme, expression level of this lncRNA has been positively correlated with the presence of infiltrating CD8+ T cell population. However, its expression level in this type of tumor has been negatively correlated with the presence of infiltrating dendritic cell population. In low-grade glioma, MEG3 expression has been negatively correlated with the amounts of infiltrating B, CD8+ T and CD4+ T cell populations as well as macrophage, neutrophil, and dendritic cells^30^. MEG3 is also involved in the process of peripheral nerve injury. Expression of this lncRNA has been found to be up‐regulated after sciatic nerve injury. Down‐regulation of MEG3 in Schwann cells has improved proliferation and migration of these cells^31^. Thus, up-regulation of MEG3 in the peripheral blood of NMOSD patients might contribute to the unbalanced immune responses in these patients in terms of over-activation of B and T cells. Similarly, up-regulation of NEAT1 in these patients is probably involved in the activation of immune responses, since this lncRNA has been shown to promote activation of inflammasomes in macrophages^32^. The role of TUG1 in the pathobiology of NMOSD should be assessed in future, since it is a possible modulator of Siglec-15-related anti-immune activities^33^.

Notably, our study revealed no significant difference in the expression of mentioned lncRNAs between female and male NMOSD patients. This finding shows the similar effects of these lncRNAs in the pathogenesis of NMOSD between males and females.

TUG1 and PANDAR expression levels were positively correlated with age and age at onset. Thus, it is possible that expression levels of these two lncRNAs are affected by disease course and reflect the pathogenic effects of NMOSD. However, we could not detect any correlation between their expression and EDSS scores either at the initial phase of the disorder or at the point that blood samples were collected. On the other hand, NEAT1 expression level was negatively correlated with EDSS at the start point.

MEG3 had an ideal performance in the separation of NMOSD cases from healthy controls. Other lncRNAs could also efficiently separate NMOSD cases from controls with appropriate AUC values. Cumulatively, NEAT1, PANDAR, MEG3 and TUG1 lncRNAs can be considered as appropriate disease markers for NMOSD. For better assessment of the role of these lncRNAs in the pathobiology of NMOSD, we suggest assessment of their expression in different sets of blood cells, including peripheral blood mononuclear cells.

### Supplementary Information

Supplementary Information.

Supplementary Table S1.

## Data Availability

All data generated or analyzed during this study are included in this published article [and its supplementary information files].
