# Supplementary material for: Significant up-regulation of lncRNAs in neuromyelitis optica spectrum disorder
Source: Sci Rep. 2023 Oct 31;13:18692. doi: 10.1038/s41598-023-45457-w (PMC10618193; doi:10.1038/s41598-023-45457-w)
Supplement: Supplementary file 2 — Supplementary Table S1. [file 41598_2023_45457_MOESM2_ESM.docx]

Table S1. Primer sequences.

| Product length | Primer and probe length | Primer and probe sequences | Gene name |
| --- | --- | --- | --- |
| 78 | 20 | F: CCAGTGTGAGTCCTAGCATTGC | *NEAT1* |
|  | 22 | R: CCTGGAAACAGAACATTGGAGAAC |  |
| 149 | 24 | F: ACCGGAGGAGCCATCTTGTC | *TUG-1* |
|  | 24 | R: GAAAGAGCCGCCAACCGATC |  |
| 81 | 24 | F: GTTTTCCTGTTCGTCGATTCTGG | *PANDA* |
|  | 23 | R: GGAAAGCTGAGAGAGACTTTGAAC |  |
| 111 | 18 | F: TGGCATAGAGGAGGTGAT | *MEG3* |
|  | 19 | R: GGAGTGCTGTTGGAGAATA |  |
